# Complementary and alternative medicine use among diabetic patients in Africa: a Kenyan perspective

**DOI:** 10.11604/pamj.2013.15.110.2925

**Published:** 2013-07-25

**Authors:** Duncan Mwangangi Matheka, Alessandro Rhyll Demaio

**Affiliations:** 1 Department of Medical Physiology, University of Nairobi, Kenya; 2 Young Professionals Chronic Disease Network; 3 Copenhagen School of Global Health, University of Copenhagen; 4 Harvard Global Equity Initiative, Harvard Medical School, USA

**Keywords:** Traditional medicine, herbal medicine, dietary supplements, CAM, diabetes mellitus, regulation, integration, Kenya, developing countries

## Abstract

Complementary and alternative medicine (CAM) use is common among patients with chronic diseases in developing countries. The rising use of CAM in the management of diabetes is an emerging public health concern given the potential adverse effects, drug interactions and benefits associated with its use. Herbal medicine, dietary supplements, prayers and relaxation techniques are some of the most frequently used CAM modalities in Kenya. Cited reasons for CAM use as adjuvant therapy include dissatisfaction and inaccessibility of allopathic medicine, and recommendations by family and friends. This article explores the pattern of CAM use in Kenya and other developing countries. It also identifies some constraints to proper CAM control, and offers suggestions on what can be done to ensure safe and regulated CAM use.

## Introduction

The prevalence of diabetes mellitus (DM) worldwide is projected to rise to 552 million (representing 10% of the global adult population) by 2030 up from 366 million in 2011 [1]. The burden is worse in the developing world which represents over 80% of cases [1, 2].

In DM management, lifestyle measures, oral glucose-lowering drugs and insulin are the conventional therapies. The latter two are, however, expensive or even unavailable to many patients in developing countries [3], and are sometimes associated with adverse effects [4]. Consequently, some patients opt for complementary and alternative medicine (CAM) to manage their DM. The prevalence of CAM use among people living with DM is estimated to be as high as 80% in Africa [5, 6].

## Pattern of CAM use in Africa

Commonly used CAM therapies among diabetic patients in Africa include herbal medicines, nutritional products, spiritual healing and relaxation techniques [7–10]. These CAM therapies are extensively used by patients as adjuvant or as replacement treatment to the conventional prescribed drugs [11–14]. CAM use in Africa is amplified by the presence of traditional healers, with estimates of one traditional healer present to every 200 people [15]. These traditional healers make selective use of CAM, biomedical knowledge and language to enhance the perceived effectiveness of their treatments [15].

The use of CAM in Africa has been associated with cultural beliefs, age of patient, duration of DM, degree of complications, and advice from family and friends [16, 17]. Most importantly, the inaccessibility and shortcomings in conventional healthcare provision in Africa contribute to the high use of CAM [3].

A major concern is that diabetic patients may replace clinically proven conventional diabetes treatments with CAM agents [18, 19]. These patients rarely disclose their CAM practices to their health care providers (HCPs) [20], an issue which warrants particular attention. There is a potential risk of drug interaction when these agents are used as adjuvants to allopathic medicine. They may also interfere negatively with glycemic control, and cause adverse effects and additional complications [6, 15, 19, 21]. It is a well-known fact that most CAM agents contain active ingredients for which appropriate doses and side effects have not been determined. They are therefore likely to be administered at inconsistent doses, with the potential for fatal health effects and mortalities [17].

## Challenges in controlling CAM in Africa

A number of constraints exist in the control of CAM use in Africa. For instance, there is lack of integration of CAM therapies into African mainstream health care systems. This is despite the World Health Organization (WHO) recommendation to “integrate traditional and CAM therapies into national health care systems” [6].

Another major concern is the lack of regulation on CAM use in Africa and other developing countries, and therefore exposing the population to potential harm. There exists limited quality assurance with most CAM regulatory processes falling outside the scope of most government drug and therapeutic agencies in Africa. For instance, the registration of herbalists in Kenya is done by the Ministry of Social services, but in essence most of the traditional herbalists are not even aware of this.

There is also limited research on CAM use by people with diabetes in developing countries including Kenya. Some CAM products may also be beneficial and safe; but the lack of randomized controlled trials makes their use controversial [21].

HCPs are also not aware that so many of their diabetic patients use CAM therapies. HCPs should therefore have this in mind, and routinely take a thorough history to document any such therapies and discuss these practices with their patients in order to safeguard their health. HCPs should educate their patients on the importance of adherence, controlling blood sugars and avoidance of potentially dangerous CAM.

## Conclusion

CAM is widely used among diabetic patients as an adjunct to conventional therapy in developing countries. This could result in ineffective diabetes management and cause adverse effects, especially since the CAM use is rarely disclosed to HCPs. Empirical evidence, integration and stringent national regulatory safe-guards should guide the safe and appropriate CAM use and sales. Legislation to govern CAM use is therefore necessary and inevitable. Above all, conventional medications should be easily accessible. HCPs should also be aware of CAM use, and educate their patients accordingly. There is a need for urgent multi-sectorial action to streamline CAM use among patients in Africa and other developing countries.
